# Imported case of cholera from India: the first recorded in Bulgaria in over a century

**DOI:** 10.1093/omcr/omaf113

**Published:** 2025-07-27

**Authors:** Ralitsa Yordanova, Eugeni Pentchev

**Affiliations:** Department of Infectious Diseases, Parasitology and Tropical Medicine, Medical University Sofia, North Pavilion, bul. “akad. Ivan E. Geshov” No. 17, 1431, Sofia, Bulgaria, Bulgaria; Department of Infectious Diseases, Parasitology and Tropical Medicine, Medical University Sofia, North Pavilion, bul. “akad. Ivan E. Geshov” No. 17, 1431, Sofia, Bulgaria, Bulgaria

**Keywords:** cholera, non-endemic, imported case

## Abstract

Cholera is an acute bacterial infection characterized by profuse watery diarrhea, vomiting, and dehydration, potentially leading to severe complications and death. It remains endemic in regions with poor sanitation and limited access to clean water, particularly in South and Southeast Asia and parts of Africa. In Europe, only sporadic cases are reported annually, and are typically imported by travelers returning from endemic areas. Mild cases often present with non-specific gastrointestinal symptoms, making them clinically indistinguishable from other enteric infections. To initiate appropriate epidemiological measures and prevent transmission in non-endemic regions, cholera should be considered in any case of acute watery diarrhea in individuals with a recent travel history to endemic areas. We report a case of cholera in a patient returning from India—the first recorded in Bulgaria in over a century.

## Introduction

Cholera is an acute bacterial infection that typically presents with watery diarrhea, vomiting, and dehydration, and can lead to severe complications and death. The infection is caused by *Vibrio cholerae*, a Gram-negative, motile bacterium characterized by two antigens: H and O. Based on the O antigen, *V. cholerae* is divided into several serogroups, among which O1 and O139 are associated with the pandemic spread of the disease. *V. cholerae* O1 is further subdivided into two biotypes: classical and El Tor [1].

Since 1817, when the first pandemic was described, seven cholera pandemics have been recorded. The first six originated in the Indian subcontinent and were caused by the classical biotype of *V. cholerae* O1. The seventh pandemic, which continues to the present day, began in 1961 in Indonesia and is caused by the El Tor biotype of *V. cholerae* O1. It has spread across five continents and has affected more countries than any previous pandemic.

In 1992, a new serogroup, *V. cholerae* O139, emerged and has since become endemic in at least 11 countries.

This bacterium is typically found in coastal waters that are contaminated with human waste. Cholera is transmitted via the fecal-oral route. Infection occurs through the consumption of contaminated water or food—especially seafood from affected areas or through direct person-to-person contact [2].

The symptoms of cholera include watery diarrhea, described as ‘rise-water stools’ and vomiting. Fever is not a typical symptom. Severe cases, which account for approximately 10% of infections, are characterized by profuse watery diarrhea and vomiting that lead to significant dehydration and electrolyte loss. This can result in hypovolemic shock and death if not promptly treated. However, in most cases, the disease presents with mild symptoms that are indistinguishable from other gastrointestinal infections [2].

Cholera is endemic to regions with poor sanitation and limited access to clean water, particularly in South and Southeast Asia and Africa [1–3].

## Case report

A 23-year-old man presented with a 7-day history of watery diarrhea, which initially occurred 8–9 times per day, gradually decreasing to 6 times per 24 h by the day of hospital admission. He reported increased bowel movements, but denied experiencing abdominal pain or fever. The onset of symptoms coincided with his return from India. The day prior, he had been in New Delhi, India, where he had consumed suspicious street food, specifically meat. After his return to Bulgaria, he underwent an outpatient microbiological stool test due to his symptoms. The result was positive for *V. cholerae*, and the patient was referred for hospitalization. He had no other known medical conditions. By the day of hospital admission, the patient was in a mildly impaired general condition with signs of mild dehydration, including a coated tongue and hypohydrated mucosae. His blood pressure was 117/73 mmHg, and heart rate was 96 bpm. Auscultation revealed increased bowel sounds. There were no pathological findings from other organs or systems upon examination. The results from laboratory blood and urine tests are shown in Table 1.

**Table 1:** Laboratory indicators by days.

	Illness day 7, Hospital day 1	Illness day 8, Hospital day 2	Illness day 11, Hospital day 5	Reference range
Haematology				
WBC count	9.59			3.50–10.50 x 10^9^/L
Neutrophils	79.5			50.00–80.00%
Lymphocytes	18.9			3.50–10.50%
Monocytes	1.6			3.50–12.20%
RBC count	5.71			4.50–6.20 x 10^12^/L
Haemoglobin	169			120–180 g/l
Platelet count	314.0			140.0–440.0 x 10^9^/L
Biochemistry				
CRP	19.37			0.0–10.00 mg/l
Blood glucose	5.7	5.2	5.7	3.33–6.40 mmol/l
Urea	4.8			2.1–7.1 mmol/l
Creatinine	80.25			61–104 μmol/l
AST	30			2–38 U/L
ALT	19			2–41 U/L
Lactate		0.9		0.5–2.2 mmol/l
Chloride	114.0	115.0	110.0	96–106 mmol/l
Potassium	2.7	3.1	4.0	3.6–5.6 mmol/l
Sodium	138.0	139.0	140.0	136.0–151.0 mmol/l
Blood gases				
pH	7.332	7.303	7.392	7.360–7.440
pCO_2_	30.8	35.9	42.8	35.0–48.0 mmHg
HCO_3_ act	15.9	17.3	25.5	21–25 mmol/l
HCO_3_ stat	17.8	18.1	24.9	21–25 mmol/l
BE ecf	−8.9	−7.9	1.1	−2.5 – +2.5 mmol/l
Urine				
pH		6.0		5.0–7.0
SG		1.015		1.001–1.026
Bilirubin		Neg		0.05–3.2
Urobilinogen		Norm		0.0–1.7
Ketones		Neg		0.0–0.0
Ascorbic acid		Neg		
Glucose		Norm		0.0–0.84
Protein		Neg		0.0–0.15
Erythrocytes		Neg		0–5
Leucocytes		Neg		0–5
Nitrites		neg		0.0–0.0

*V. cholerae* O1 was isolated from the patient’s stool using specific culture media—thiosulfate citrate bile salts sucrose (TCBS) agar. Antibiotic sensitivity testing revealed that the bacteria were sensitive to cefuroxime, ceftriaxone, amikacin, doxycycline, and levofloxacin, but resistant to erythromycin, penicillin, and trimethoprim-sulfamethoxazole.

The patient was treated with intravenous fluids, including Ringer lactate solution and Serum glucose 5% (2500 ml on the first day, then gradually reduced) and 15% potassium chloride (30 ml on the first day, then reduced). Oral rehydration solution (ORS) was also administered. Additionally, amikacin 500 mg was given intravenously twice daily for five days.

The patient was discharged after three consecutive days of negative stool cultures for *V. cholerae* (hospital days 7, 8, and 9) with clinical and laboratory improvement.

## Discussion

Cholera is not considered endemic in Europe, and only sporadic cases are reported annually, typically imported by travelers returning from endemic regions. For instance, no cases were reported in Europe in 2020, and only two cases were documented in 2021 [1–5]. In Bulgaria, the last recorded case dates back to 1921, making the current case the first in over a century.

The increased accessibility of international air travel and the resurgence of global mobility following the COVID-19 pandemic have elevated the risk of importing cholera cases into non-endemic countries. According to the World Health Organization (WHO), cholera should be considered in any case of acute watery diarrhea in individuals returning from endemic areas [6].

While rehydration remains the cornerstone of cholera treatment, WHO recommends the use of antibiotics in severe cases. The International Centre for Diarrhoeal Disease Research (ICDDR) advises antibiotic use in both moderate and severe dehydration [7]. Antibiotic therapy has been shown to reduce the duration of illness, accelerate recovery, and decrease the period of bacterial shedding [7, 8].

Prompt recognition and early diagnosis are essential for initiating appropriate epidemiological measures to prevent further transmission. In the context of increased travel and global interconnectedness, heightened awareness and clinical suspicion are critical, especially in non-endemic regions like Bulgaria.

## Consent

The patient gave written consent for publication of this case.

## Guarantor

Dr. Ralitsa Yordanova, Dr. Eugeni Pentchev.

## References

[1] Steffens I, European Centre for Disease Prevention and Control. Cholera. In: ECDC. Annual epidemiological report for 2021. Stockholm: ECDC; 2023. Available at: https://www.ecdc.europa.eu/sites/default/files/documents/cholera-annual-epidemiological-report-2021.pdf

[2] Steffens I , European Centre for Disease Prevention and Control (ECDC) The European Surveillance System (TESSy): Surveillance Atlas of Infectious Diseases. [(accessed on 15 April 2025)]. Available online: https://atlas.ecdc.europa.eu/public/index.aspx

[3] Asantewaa AA, Odoom A, Owusu-Okyere G. et al. Cholera outbreaks in low- and middle-income countries in the last decade: a systematic review and meta-analysis. Microorganisms*.* 2024;12:2504. 10.3390/microorganisms12122504 39770707 PMC11728267

[4] Steffens I , European Centre for Disease Prevention and Control (ECDC). Communicable disease threats report CDTR. Week 47, 21-27 November 2021. Available at: https://www.ecdc.europa.eu/sites/default/files/documents/communicable-disease-threats-report-week%2047-2021.pdf

[5] Steffens I, European Centre for Disease Prevention and Control (ECDC) . Introduction to the Annual Epidemiological Report. In: ECDC. Annual epidemiological report. Stockholm: ECDC. Available at: https://www.ecdc.europa.eu/en/surveillance-and-disease-data/annual-epidemiological-reports/introduction-annual

[6] Global Task Force on Cholera Control (GTFCC) Interim Guidance Document on Cholera Surveillance 2024 Global Task Force on Cholera Control (GTFCC) Surveillance Working Group. WHO. Available online: https://www.gtfcc.org/wp-content/uploads/2025/04/public-health-surveillance-for-cholera-guidance-document-2024.pdf

[7] Nelson EJ, Nelson DS, Salam MA. et al. Antibiotics for both moderate and severe cholera. N Engl J Med 2011;364:5–7. 10.1056/NEJMp1013771 Epub 2010 Dec 9 21142691

[8] Leibovici-Weissman Y, Neuberger A, Bitterman R. et al. Antimicrobial drugs for treating cholera. Cochrane Database Syst Rev 2014;2014:CD008625. 10.1002/14651858.CD008625.pub2 24944120 PMC4468928

